# Medicine as a Profession

**Published:** 1897-09-04

**Authors:** 


### Medicine as a Profession.

The annually recurring period of the commencement of study at the great medical schools of London or of the provinces is one which cannot fail to bring some anxiety to the parents of the young men who have selected medicine as a career, and whose future must largely depend upon the degree in which the choice thus made has been a judicious one. Alone among the followers of human occupations the professors of medicine are engaged in an unceasing battle against the injurious influences by which they live, and in striving to prevent the evils which they are paid to cure. They are forbidden, by the written or unwritten laws of their corporations, to conceal any truth which may benefit mankind, or to make it a matter of personal gain as a monopoly. Everything which they discover, and almost everything which they invent, becomes at once the property of their competitors and of the whole community; and the knowledge which has been laboriously acquired by physicians and surgeons too often furnishes a basis for the impostures of quacks, or is seized upon by the followers of some other calling, in order that it may by them be turned to profitable account. The general result of this course of conduct, pursued unremittingly over many years, has been that disease is much less prevalent than it was even within the recollections of people not very far advanced in life, and that it is more under control than it has been at any former period, in the sense, especially, that the duration of nearly every common illness has been curtailed. There is therefore, at least in some directions, less work for doctors to do; while, at the same time, the census returns do not point to any diminution in the proportionate numbers of those by whom it is to be done. In these circumstances parents and guardians must often feel much doubt as to the wisdom of the choice made by the young people in whom they are interested, and may reasonably ask what prospects the profession will offer them. Is there reasonable ground for believing that it will afford an honest living, in the social position of a gentleman?

Sir James Paget, some years ago, traced out the careers of one thousand medical students who had entered at St. Bartholomew's Hospital, and who had left it at least 15 years before the commencement of his inquiry. The results, although very interesting, are too long for detailed quotation in this place. It is evidently a necessary condition of success in any profession that it should be followed for a sufficient time; and hence it is necessary to omit from Sir James Paget's list all who turned early away to other occupations, or who died either before or shortly after they became qualified. By doing this the total number is reduced from 1,000 to 776, out of whom only 56, or 7·2 per cent., are recorded to have failed entirely; 124, or 16 per cent., had "very limited" success; and the remaining 596, or 76·8 per cent., obtained respectable positions and comfortable maintenance, increased, in the cases of 66, or 8·5 per cent., to "considerable," and in the cases of 23, or 3 per cent., to "distinguished" success. It is not probable that any great change in the figures would be the outcome of a similar investigation made to-day; and, if this be conceded, and if we think of the proportion borne by briefless barristers to Queen's Counsel, by poor curates to bishops and deans, by qualified solicitors who do not rise above paid clerkships to their more fortunate brethren, by the lower grades of commissioned officers in the army and navy to those who rise high enough to retire upon comfortable pensions, it must probably be admitted that medicine fully holds its own in the race of life, and that it may be embraced by any young man possessed of fair abilities and of a reasonable degree of energy and perseverance, as a calling in which, if he fails, his failure will be due to faults or shortcomings of his own, rather than to any circumstances peculiar to the profession which he has chosen. It is highly probable that, by the development of the public health service and in other ways, a variety of compensations for the diminished demands upon the family practitioner have come into existence, and that new openings have been made as the older ones have become somewhat narrowed. Nevertheless, the consideration of ways and means is as important in the medical profession as in any other. The young doctor has to live while he is making his way; and, if he is to live with clean hands, he must have command of a certain amount of capital. The more he has, comparatively speaking, the more free will he be to select the line of work best suited to him; but in medicine, as in other vocations, enough money to weaken ordinary motives for exertion is more likely to lead to failure than to reputation. On the other hand, no error is more certain to entail disastrous consequences than that of engaging in practice of any kind without sufficient means to wait a proper and reasonable time for returns. A young man who is tempted to take this course can hardly do better than look at the question through the spectacles of a very keen observer. He should read George Eliot's "Middlemarch," and should ponder over the lesson which it conveys.

